# Sensor Fusion of Cameras and a Laser for City-Scale 3D Reconstruction

**DOI:** 10.3390/s141120882

**Published:** 2014-11-04

**Authors:** Yunsu Bok, Dong-Geol Choi, In So Kweon

**Affiliations:** Robotics and Computer Vision Lab., KAIST, 291 Daehak-ro, Yuseong-gu, Daejeon 305-701, Korea; E-Mails: ysbok@rcv.kaist.ac.kr (Y.B.); dgchoi@rcv.kaist.ac.kr (D.-G.C.)

**Keywords:** sensor fusion, structure from motion, 3D reconstruction

## Abstract

This paper presents a sensor fusion system of cameras and a 2D laser sensor for large-scale 3D reconstruction. The proposed system is designed to capture data on a fast-moving ground vehicle. The system consists of six cameras and one 2D laser sensor, and they are synchronized by a hardware trigger. Reconstruction of 3D structures is done by estimating frame-by-frame motion and accumulating vertical laser scans, as in previous works. However, our approach does not assume near 2D motion, but estimates free motion (including absolute scale) in 3D space using both laser data and image features. In order to avoid the degeneration associated with typical three-point algorithms, we present a new algorithm that selects 3D points from two frames captured by multiple cameras. The problem of error accumulation is solved by loop closing, not by GPS. The experimental results show that the estimated path is successfully overlaid on the satellite images, such that the reconstruction result is very accurate.

## 1. Introduction

Reconstructing three-dimensional structures is a fundamental problem in the area of computer vision. Reconstructed models are useful in various applications, such as navigation, simulation and virtual reality. The most popular sensors to obtain 3D structures are CCD cameras and laser sensors. Recently, image-based methods have shown impressive results in terms of accuracy and scaling, owing to the improvement in computing devices and techniques related to structure-from-motion (SFM) approaches and bundle adjustment [1]. For example, Snavely *et al.* [2] collected images from the Internet and reconstructed 3D structures of several tourist attractions. The final result from a huge amount of data is obtained by utilizing parallel computing resources [3] and visualized via multi-view stereo [4]. Pollefeys *et al.* [5] used multiple cameras with a small degree of overlap and utilized a GPU to implement a plane sweeping method. However, camera-based methods have common limitations. The depth of a feature point computed by triangulation is not highly accurate unless it is seen in various directions and matched correctly. For this reason, only stable features are reconstructed accurately, while homogeneous areas cannot be reconstructed without assumptions. Three dominant planes (Manhattan world) [5,6] and vertical walls [7] are reasonable assumptions when reconstructing urban or indoor scenes, but they are not suitable for general scenes.

Laser sensors provide an accurate depth of their field of view without triangulation. Howard *et al.* [8] and Frueh *et al.* [9] used 2D laser sensors to reconstruct urban scenes. The range data scanned by the vertical sensors are accumulated based on the localization result computed by the horizontal sensor. Smith *et al.* [10] focused on compressing accumulated laser scans into a small number of meshes. Fentanes *et al.* [11] analyzed 3D data scanned by rotating vertical laser sensor for outdoor navigation and reconstruction. Banno *et al.* [12] obtained range data using 3D laser sensors and 2D laser sensors mounted on sliding modules. Xiao and Furukawa [13] detected lines from the point cloud obtained by laser sensors and merged them into a plane-based 3D model. The only process required for 3D reconstruction using laser sensors is the registration of the local range data. Registering data from 3D laser sensors is relatively easy, because we can obtain data with enough overlap. For example, Allen *et al.* [14] extracted planes and lines to compute a proper transformation between two 3D scans. Since the frame rate of 3D laser sensors is lower than that of 2D laser sensors, the structures scanned by 3D sensors are distorted more than those scanned by 2D sensors if they are mounted on a fast-moving ground vehicle. The high price of 3D laser sensors is also a problem of using them. If scanned data by 2D laser sensors can be accumulated accurately, using 2D laser sensors is a better solution to several applications than using 3D sensors.

Fusion of different sensors is a solution to the problems mentioned above. Sensors have different characteristics, strengths and weaknesses. Utilizing multiple sensors in a system can complement their weaknesses each other. A combination of cameras and laser sensors is a popular example of sensor fusion. It has been applied to the 2D-based localization of robots in indoor environments [15–18] and outdoor applications [19,20]. Recently, RGB-D sensors, such as Kinect, became popular in the robotics community. They are utilized in various research issues: SLAM [21–23], photometry [24–26], recognition [27,28] and other applications [29].

Bok *et al.* [30,31] proposed a new concept of camera-laser fusion for 3D reconstruction. The accurate depth provided by the laser sensor is independent of the pose of the system and the target scenes (even homogeneous areas can be scanned). The motion of the 2D laser sensor in 3D space (including an absolute scale) is computed by utilizing the scanned points projected onto the images. The system proposed in [30] is capable of reconstructing arbitrary scenes without any assumptions. However, it is not appropriate for urban scenes, because it is designed to be carried by a human operator to reconstruct narrow scenes. It does not work well if the target objects are distant or the system moves rapidly. Recently, Moghadam *et al.* [32] developed a hand-held system, which consists of a 2D laser sensor, camera and IMU for 3D reconstruction. It also has problems in large-scale outdoor environments, similar to the system by Bok *et al.* [30].

In this paper, we propose a sensor fusion system and algorithms for large-scale 3D reconstruction. The system is designed to be mounted on a ground vehicle to reconstruct scenes with roads. A vertical 2D laser sensor scans structures, and the reconstruction is done by accumulating the data scanned by the sensor. The difference between the proposed method and previous ones involving the use of two 2D laser sensors is that we do not assume 2D motion, but estimate motion in 3D space. Other researches utilizing vertical laser sensors tried to overcome the 2D assumption [33], but they are not completely free from ‘ground vehicle movement’. In this paper, the motion of the system is estimated accurately even if the system moves freely in 3D space, so that it may be carried by any types of platforms, such as human operators or helicopters. Moreover, a high frame rate and good synchronization allow the system to be mounted on a fast-moving ground vehicle, whereas the previous system [30] required being carried at a low speed. We present new methods of estimating frame-by-frame motion and reducing accumulated error by utilizing a few closed loops. The results show that the motion is very accurate over thousands of frames and over tens of kilometers using several closed loops without GPS.

This paper is an extended version of [34]. The motion estimation algorithm (described in Section 3) of the proposed system is published in [35]. The performance of the algorithms in this paper is verified by several quantitative analyses and experiments using real data.

## 2. Sensor System

### 2.1. 3D Reconstruction Using Range Sensors

**Figure 1.**
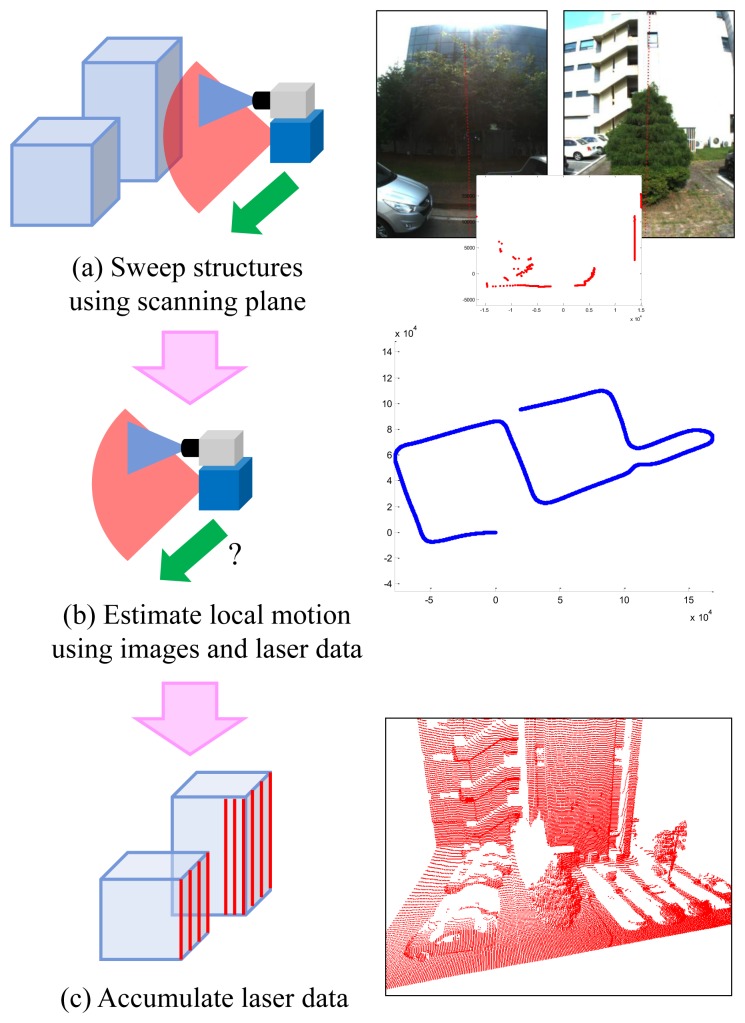
Using laser sensors, 3D structures are reconstructed via three steps: (**a**) capture data; (**b**) estimate motion and (**c**) accumulate scans. The only problem is to estimate the motion of the system as accurately as possible.

In this paper, 3D structures are reconstructed by accumulating vertical laser scans (see Figure 1). This methodology looks old-fashioned compared to 3D sensors, such as Velodyne, time-of-flight camera or Kinect. However, 2D-laser-based reconstruction is still a cheap and efficient solution. Using any range sensor, 3D reconstruction requires its pose in 3D space. Moreover, any range sensor with known poses can be used for 3D reconstruction. In this approach, the only problem is the motion estimation of the sensor. Usually, 3D sensors do not require additional sensors for motion estimation: 3D-3D registration is enough for that. However, few sensors are appropriate for outdoor reconstruction. For example, Kinect [36] works only in an indoor environment, because the infrared pattern from a built-in projector is not recognized well in daylight condition. Time-of-flight cameras [37,38] provide low-resolution range images from a narrow field of view. Velodyne [39] provides high-quality information in outdoor environments, but it is relatively expensive.

We use a vertical 2D laser sensor to obtain 3D information. It is impossible to estimate the horizontal motion of a vertical laser sensor using only scan data from it, because adjacent scans have no relation (overlap). Usually, additional sensors are attached to estimate the motion of the vertical laser sensor. As we mentioned in Section 1, a number of research using vertical laser sensors has been published. We utilize a number of CCD cameras to estimate the motion of the sensor.

### 2.2. Sensor Configuration

**Figure 2.**
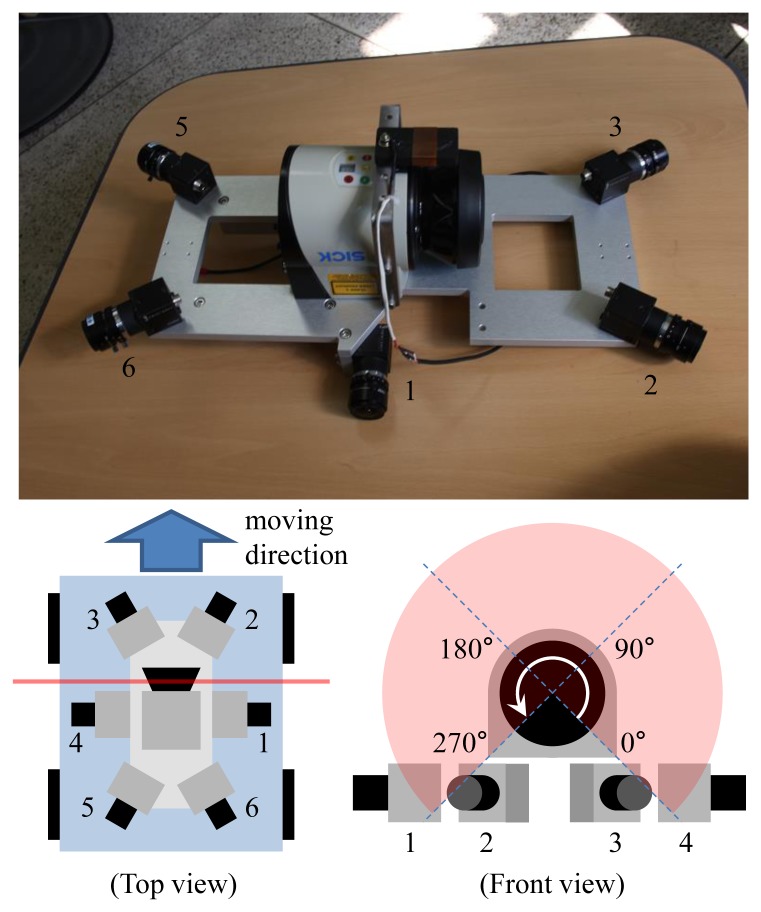
The proposed sensor fusion system contains six cameras and one 2D laser sensor. The vertical laser sensor scans the structures. The non-overlapping cameras provide a wide field of view, which makes the estimated motion accurate.

**Figure 3.**
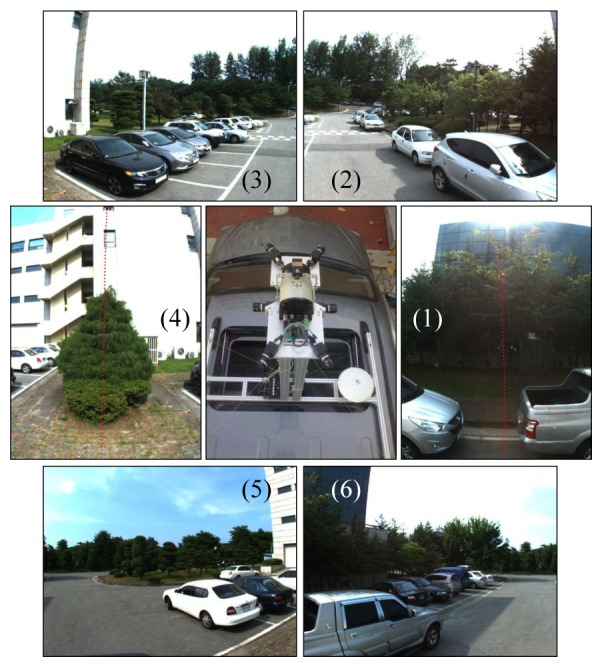
An example of the data captured by the proposed vehicle-mounted system : The center image is the top view of the system mounted on a ground vehicle. The other six images are captured by six non-overlapping cameras. The laser points are projected onto Cameras 1 and 4.

Our previous system [30] consists of four cameras and two 2D laser sensors. We did not have to pay attention to the synchronization issue, because the movement of the system carried by a human operator was slow. However, the synchronization issue becomes serious if the system is mounted on a ground vehicle moving at a high speed. Since we cannot adjust the angular velocity of the mirror in a 2D laser sensor, two laser sensors cannot be synchronized if there is a slight difference between their scanning speeds. We decided to limit the use of laser units to one to avoid the synchronization issue. Figure 2 shows the proposed sensor system, which consists of six cameras and one 2D laser sensor. The laser sensor (SICK LMS151) scans 270 degrees, and the center (135 degrees) of the angular range is headed toward the sky, such that the laser sensor scans both sides of the vehicle. Two cameras (1 and 4 in Figure 2) see the 225- and 45-degree directions of the laser's scanning angle. Most laser data measured at the interval of 180–270 degrees and 0–90 degrees are projected onto Cameras 1 and 4, respectively. These cameras are rotated 90 degrees (roll) to project as many laser points onto them as possible. The other four cameras track only image features. We avoided the forward and backward heading directions, because there are usually few static features in those directions in a scene with roads. Figure 3 shows an example of the data captured by the system.

### 2.3. Synchronization

The capturing speed of the laser sensor is 50 fps, while that of the camera is 60 fps. This difference makes the sensors capture data at different moments, while also making the calibration result useless. In order to prevent this problem, we develop a synchronization system for one laser sensor and multiple cameras. The laser sensor used in the proposed system does not give information regarding its shooting time; therefore, an infrared detector is attached onto the front of the laser sensor. The detected laser signal is sent to every camera after passing through a noise filter and an amplifier. The trigger signal is generated slightly (0.4 ms) after the infrared ray is received, due to hardware processing time.

## 3. Motion Estimation

### 3.1. Laser Points as 3D Points

**Figure 4.**
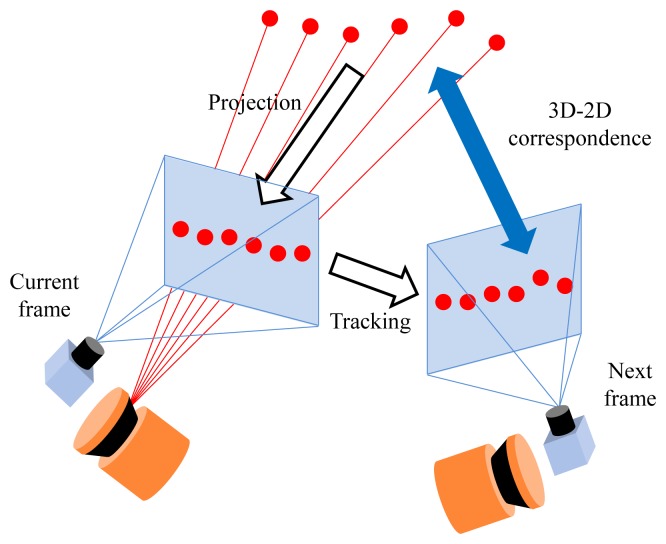
Using the camera-laser calibration result, the laser points can be transformed into the camera coordinate and, thus, can be projected onto the image. Tracking them on the image generates 3D-2D correspondences, which are useful for the motion estimation of the system.

In the motion estimation process, the most important difference between our method and typical camera-based methods is that we have additional laser points with known depths. The system is fully calibrated using a planar pattern (cameras [40]) and the point-line constraint (between cameras and laser sensor [30]). The points scanned by the laser sensor can be transformed into the camera coordinate and projected onto the images. We track those points on the images to obtain 3D-2D correspondences, which are useful in the motion estimation process (see Figure 4). Any template-based methods, such as the KLT (Kanade-Lucas-Tomasi) tracker [41], can be used to find their correspondences, but feature-based methods, such as SIFT [42] or SURF [43], cannot, because the locations of the laser points on images are different from those of feature points. Although a small number of them can be tracked properly, an advantage of using the laser points is that the motion is estimated without the previous motions, which are required to triangulate the image points.

We estimate relative pose between adjacent frames using laser points and their tracking results. RANSAC [44] is a well-known method of pose estimation using correspondences, including outliers. We sample a number of point sets—three 3D-2D correspondences for each set—and compute pose candidate for each set. The candidate with the maximum number of inliers (points with error below a user-defined threshold) is selected as an initial solution. It is refined via non-linear optimization, which minimizes projection errors of inliers (laser points and image features). Laser points and image features are tracked through multiple frames (5 and 20 frames for laser points and image features, respectively, in our experiments). All projection errors of inliers in tracked frames are minimized in the optimization process. Bundle adjustment [1] may be applied to the final result, but it must be modified to include laser points. A modification of bundle adjustment for sensor fusion system is proposed in [31].

**Figure 5.**
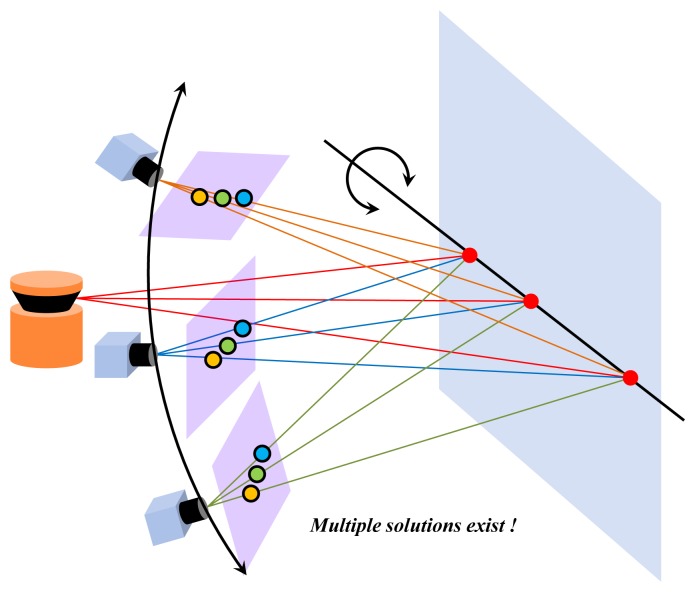
Scanning a plane with a 2D laser sensor leads to degeneration when using the typical three-point algorithms, because all laser points are collinear in 3D space. Collinear points are not changed by any rotation on the line that contains the points.

In order to compute an initial solution of frame-by-frame motion, we can use typical image-based algorithms, such as a perspective three-point algorithm [45], for single-camera-based systems or a generalized three-point algorithm [46] for multiple-camera-based systems. However, these algorithms experience degeneration when using three ‘collinear’ points. It is because three points on a line in 3D space is not changed by any rotation whose axis is equal to the line. Since we obtain 3D points from a 2D laser sensor, the degeneration appears frequently if a plane, such as a vertical wall or the ground, is scanned (see Figure 5).

**Figure 6.**
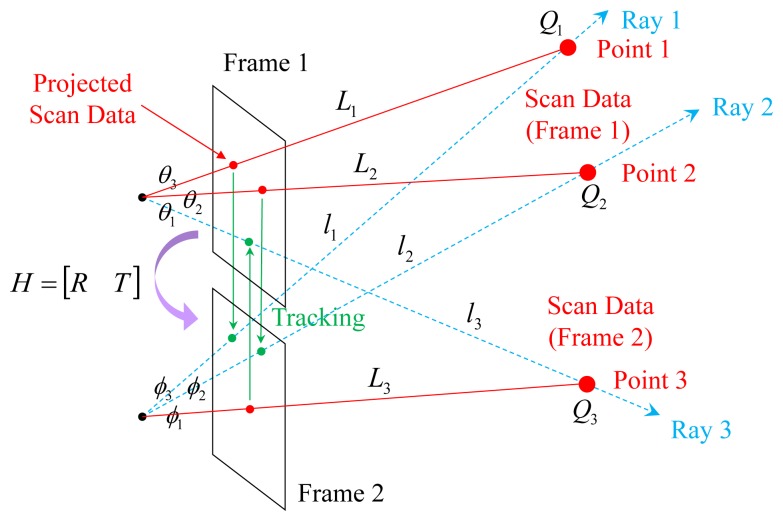
Laser three-point algorithm [47]: two points *Q*_1_ and *Q*_2_ are selected from Frame 1 and tracked to Frame 2. One point *Q*_3_ is selected from Frame 2 and tracked to Frame 1.

Bok *et al.* [47] presented the laser three-point algorithm to avoid degeneration. In order to estimate the motion between the consecutive frames, this algorithm utilizes the laser points from both frames. The union set of the laser points from both frames is expected not to be collinear if the system moves while capturing data. In Figure 6, points *Q*_1_ and *Q*_2_ scanned at Frame 1 and point *Q*_3_ scanned at Frame 2 are projected onto the corresponding images and tracked to the other frames. The angles among the rays (*θ*_1_, *θ*_2_, *θ*_3_, *ϕ*_1_, *ϕ*_2_, *ϕ*_3_*)* and the distance to the points at the scanned frame (*L*_1_, *L*_2_, *L*_3_*)* are known. The unknown variables are the distances to the points *(l*_1_, *l*_2_, *l*_3_*)* at the tracked frames. We can obtain three Equations (1)–(3) with three unknowns from Figure 6 and compute solutions by solving a four-degree polynomial equation.

(1) $$L_{1}^{2}+L_{2}^{2}-2L_{1}L_{2}\cos\theta_{3}=l_{1}^{2}+l_{2}^{2}-2l_{1}l_{2}\cos\phi_{3}$$

(2) $$L_{1}^{2}+l_{3}^{2}-2L_{1}l_{3}\cos\theta_{2}=l_{1}^{2}+L_{3}^{2}-2l_{1}L_{3}\cos\phi_{2}$$

(3) $$L_{2}^{2}+l_{3}^{2}-2L_{2}l_{3}\cos\theta_{1}=l_{2}^{2}+L_{3}^{2}-2l_{2}L_{3}\cos\phi_{1}$$

### 3.2. Generalized Laser Three-Point Algorithm

In this paper, we present a generalized version *(i.e.*, a modified one for the multiple-camera setups) of the laser three-point algorithm. Similar to the laser three-point algorithm, we assume that two points *Q*_1_ = [*x*_1_ *y*_1_ *z*_1_]*^T^* and *Q*_2_ = [*x*_2_ *y*_2_ *z*_2_]*^T^* are scanned at Frame 1 and that a point *Q*_3_ = [*x*_3_ *y*_3_ *z*_3_]*^T^* is scanned at Frame 2. *Q*_1_ and *Q*_2_ are tracked to Frame 2, and *Q*_3_ is tracked to Frame 1. The objective of the proposed algorithm is to compute the relative pose *H* = [*RT*] between two frames, which moves each point *Q_n_*(*n*=1,2,3) onto its corresponding ray with the camera center of *P_n_* = [*A_n_ B_n_ C_n_*]*^T^* and the direction vector of *V_n_* = [*a_n_ b_n_ c_n_*]*^T^*, as described in Equations (4)-(6). The ray *P_n_* + λ*_n_V_n_* on which the point *Q_n_* should lie is referred to as ‘ray *n’* in the rest of this paper.

(4) $$P_{1}+\lambda_{1}V_{1}=RQ_{1}+T$$

(5) $$P_{2}+\lambda_{2}V_{2}=RQ_{2}+T$$

(6) $$P_{3}+\lambda_{3}V_{3}=R^{T}Q_{3}+R^{T}T$$

**Figure 7.**
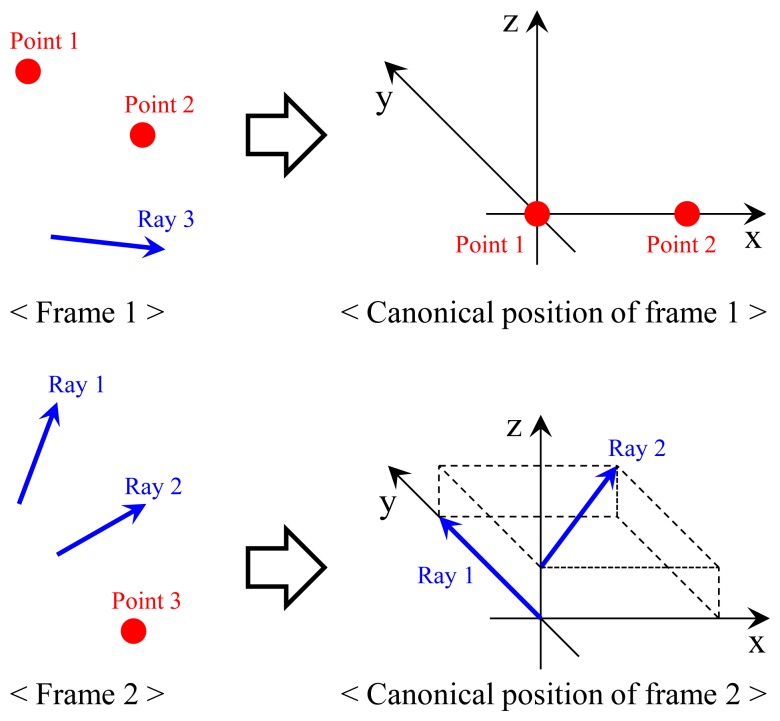
The frames are transformed into their canonical positions to simplify the following processes. In the canonical position of Frame 1, *Q*_1_ and *Q*_2_ are at the origin and on the x-axis, respectively. In the canonical position of Frame 2, Ray 1 is equal to the y-axis and Ray 2 is parallel to the x-y plane. The point on Ray 1 closest to Ray 2 is set as the origin.

**Figure 8.**
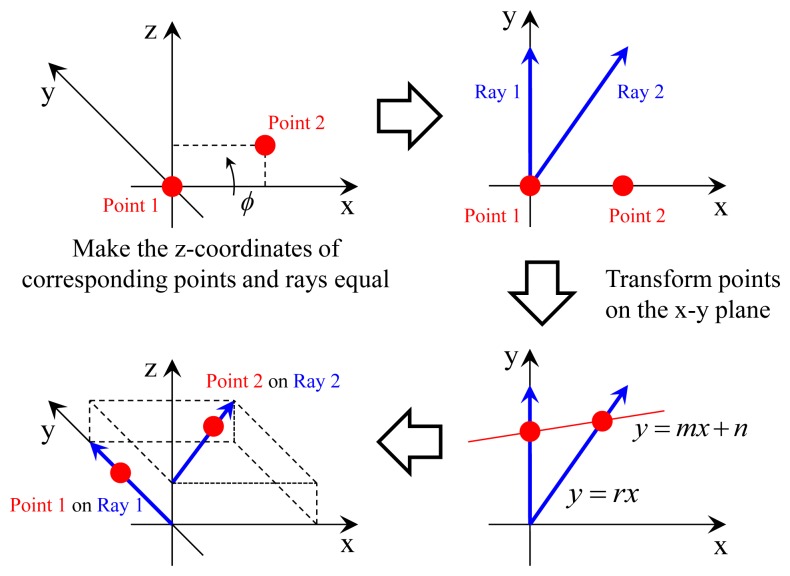
Transformation of two points onto their corresponding rays: The z-coordinates of the points are modified to be equal to their corresponding rays by a rotation on the y-axis. The points are then laid on their corresponding rays via transformation on the x-y plane.

In order to solve the problem, we compute the transformation, which makes the two points *Q*_1_ and *Q*_2_ lie on their corresponding rays via four steps. The first and the second steps are to transform Frames 1 and 2 into their canonical positions, as described in Figure 7. Without loss of generality, the canonical positions are defined to simplify following equations, which describes the process of ‘two points on two rays’ (see Figure 8). Since points *Q*_1_ and *Q*_2_ are scanned by the laser sensor, the points and the origin of the laser sensor are not collinear (unless one instance of the range data corresponding to *Q*_1_ and *Q*_2_ is equal to zero). The transformation *H*_1_ = [*R*_1_ *T*_1_] of the first step is computed using the following equations, where *O_L_* is the origin of the laser sensor in the camera coordinate (it can be computed easily using the camera-laser calibration result).

(7) $$\upsilon_{1}=\left(Q_{2}-Q_{1}\right)/\left\|Q_{2}-Q_{1}\right\|$$

(8) $$\upsilon_{2}=\left(\upsilon_{1}\times (Q_{1}-Q_{L})\right)/\left\|\upsilon_{1}\times (Q_{1}-Q_{L})\right\|$$

(9) $$R_{1}={\left[{\begin{array}{ccc} \upsilon_{1} & \upsilon_{2}\times \upsilon_{1} & \upsilon \end{array}}_{2}\right]}^{T}$$

(10) $$T_{1}=-R_{1}Q_{1}$$

In the second step, Ray 1, corresponding to point *Q*_1_, is aligned to be equal to the y-axis. The line connecting the points on the rays that are closest to the other ray is set to the z-axis. With this transformation *H*_2_ = [*R*_2_ *T*_2_], Ray 2 becomes parallel to the x-y plane. *l*_1_ and *l*_2_ are constants that make the point on Ray 1 (*P*_1_ + *l*_1_*V*_1_) closest to Ray 2 and the point on Ray 2 (*P*_2_ + *l*_2_*V*_2_) closest to Ray 1, respectively.

(11) $$\upsilon_{3}=V_{1}/\left\|V_{1}\right\|$$

(12) $$\upsilon_{4}=\left(V_{1}\times V_{2}\right)/\left\|\left(V_{1}\times V_{2}\right)\right\|$$

(13) $$R_{2}={\left[\begin{array}{ccc} \upsilon_{3}\times \upsilon_{4} & \upsilon_{3} & \upsilon_{4} \end{array}\right]}^{T}$$

(14) $$T_{2}=-R_{2}(P_{1}+l_{1}V_{1})$$

The objective of the third and the fourth step is to transform points *Q*_1_ and *Q*_2_ onto their corresponding rays (see Figure 8). Because Rays 1 and 2 are parallel to the x-y plane, we do not have to consider the z-axis if the corresponding points have proper z-coordinates. The third step is to make the z-coordinates of *Q*_1_ and *Q*_2_ equal to their corresponding rays. The z-coordinate of *Q*_1_ should be zero, and this is already satisfied. The z-coordinate of *Q*_2_ should be equal to that of *P*_2_ transformed into the canonical position of Frame 2. Let [*D_2_* 0 *d*_2_]*^T^* be the transformed coordinate of *Q*_2_*. D*_2_, *d*_2_ and the transformation *H*_3_ = [*R*_3_ *T*_3_] (*T*_3_ = 0) are computed by the following equations. *ϕ* is the rotation angle of *R*_3_.

(15) $$d_{2}=\upsilon_{4}^{T}(P_{2}-P_{1})$$

(16) $$D_{2}=\sqrt{{\left\|Q_{2}-Q_{1}\right\|}^{2}-d_{2}^{2}}$$

(17) $$R_{3}=\left[\begin{array}{ccc} \cos\phi & 0 & -\sin\phi \\ 0 & 1 & 0\\ \sin\phi & 0 & \cos\phi \end{array}\right]=\left[\begin{array}{ccc} D_{2}/\left\|Q_{2}-Q_{1}\right\| & 0 & -d_{2}/\left\|Q_{2}-Q_{1}\right\|\\ 0 & 1 & 0\\ d_{2}/\left\|Q_{2}-Q_{1}\right\| & 0 & D_{2}/\left\|Q_{2}-Q_{1}\right\| \end{array}\right]$$

The fourth step is to transform points *Q*_1_ and *Q*_2_ onto their corresponding rays in the x-y plane. Let *y* = *mx* + *n* be the equation of the line connecting the points on the rays after the transformation. The transformation changes the x-y coordinates of *Q*_1_ and *Q*_2_ in the following manner:

(18) $$Q_{1}:\left[\begin{array}{c} 0\\ 0 \end{array}\right]\to \left[\begin{array}{c} 0\\ n \end{array}\right]$$

(19) $$Q_{2}:\left[\begin{array}{c} D_{2}\\ 0 \end{array}\right]\to \left[\begin{array}{c} D_{2}/\sqrt{1+m^{2}}\\ D_{2}m/\sqrt{1+m^{2}}+n \end{array}\right]$$

Let *y* = *rx* be the equation of Ray 2 in the canonical position of Frame 2, while ignoring the z-axis. *n* can be expressed in terms of *r* and *m*, because the transformed *Q*_2_ should be on Ray 2. *M* is defined to simplify the equations.

(20) $$r=\frac{\upsilon_{3}^{T}V_{2}}{{\left(\upsilon_{3}\times \upsilon_{4}\right)}^{T}V_{2}}$$

(21) $$M\equiv \sqrt{1+m^{2}}$$

(22) $$n=\frac{D_{2}}{M}(r-m)$$

The transformation *H*_4_ = [*R*_4_ *T*_4_], which satisfies in Equations (18) and (19), is as follows:

(23) $$R_{4}=\frac{1}{M}\left[\begin{array}{ccc} 1 & -m & 0\\ m & 1 & 0\\ 0 & 0 & M \end{array}\right]$$

(24) $$T_{4}={\left[\begin{array}{ccc} 0 & n & 0 \end{array}\right]}^{T}$$

The fourth step ‘two points on two rays’ removes four degrees of freedom, while two degrees remain free. One of the free degrees is the slope *m* of the line connecting the two points mentioned in Equation (19). The other is the rotation angle *θ* of Frame 1 on the x-axis in its canonical position. The coordinates of *Q*_1_ and *Q*_2_ are not changed by this rotation, because they are on the x-axis.

**Figure 9.**
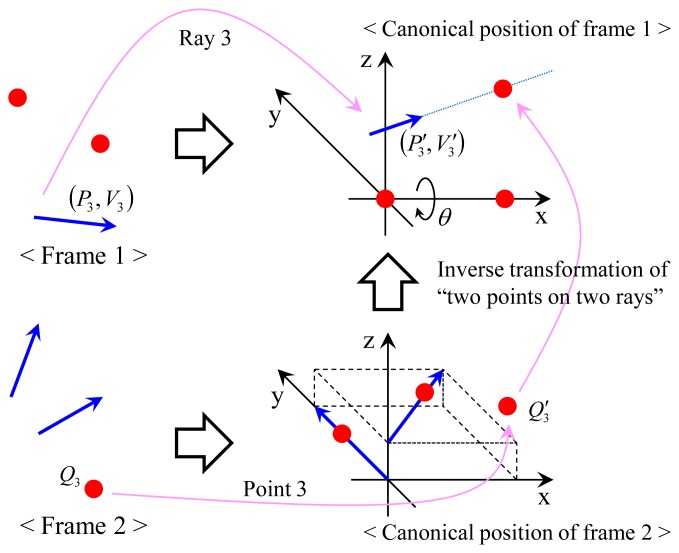
Point *Q*_3_ transformed by the inverse transformation of ‘two points on two rays’ should lie on Ray 3 (= *P*_3_ + λ_3_*V*_3_). Three equations with three unknowns are generated by this process.

We transform point *Q*_3_ to the canonical position of Frame 1 to find solutions of *m* and *θ*, as shown in Figure 9. Let $P_{3}^{\prime }={\left[\begin{array}{ccc} A_{3}^{\prime } & B_{3}^{\prime } & C_{3}^{\prime } \end{array}\right]}^{T}$ and $V_{3}^{\prime }={\left[\begin{array}{ccc} a_{3}^{\prime } & b_{3}^{\prime } & c_{3}^{\prime } \end{array}\right]}^{T}$ be the camera center and the direction vector of Ray 3, which is transformed into the canonical position of Frame 1 by *H*_1_(= [*R*_1_ *T*_1_]).

(25) $$P_{3}^{\prime }=R_{1}P_{3}+T_{1}$$

(26) $$V_{3}^{\prime }=R_{1}V_{3}$$

Let $Q_{3}^{\prime }={\left[\begin{array}{ccc} x_{3}^{\prime } & y_{3}^{\prime } & z_{3}^{\prime } \end{array}\right]}^{T}$ be point *Q*_3_ transformed into the canonical position of Frame 2 by *H*_2_(= [*R*_2_ *T*_2_]).

(27) $$Q_{3}^{\prime }=R_{2}Q_{3}+T_{2}$$

By means of the inverse transformation of *H*_4_(= [*R*_4_ *T*_4_]), *Q*_3_ is changed in the following manner:

(28) $$\left[\begin{array}{c} x_{3}^{\prime \prime }\\ y_{3}^{\prime \prime }\\ z_{3}^{\prime \prime } \end{array}\right]=R_{4}^{T}\left[\begin{array}{c} x_{3}^{\prime }\\ y_{3}^{\prime }\\ z_{3}^{\prime } \end{array}\right]-R_{4}^{T}T_{4}=\frac{1}{M}\left[\begin{array}{ccc} 1 & m & 0\\ -m & 1 & 0\\ 0 & 0 & M \end{array}\right]\left[\begin{array}{c} x_{3}^{\prime }\\ y_{3}^{\prime }-n\\ z_{3}^{\prime } \end{array}\right]=\left[\begin{array}{c} f(m)\\ g(m)\\ z_{3}^{\prime } \end{array}\right]$$

(29) $$f(m)=\frac{x_{3}^{\prime }+my_{3}^{\prime }}{M}-\frac{D_{2}m(r-m)}{M^{2}}$$

(30) $$g(m)=\frac{-mx_{3}^{\prime }+y_{3}^{\prime }}{M}-\frac{D_{2}(r-m)}{M^{2}}$$

Applying the inverse transformation of *H*_3_(= [*R*_3_ *T*_3_]),

(31) $$\left[\begin{array}{c} x_{3}^{\prime \prime \prime }\\ y_{3}^{\prime \prime \prime }\\ z_{3}^{\prime \prime \prime } \end{array}\right]=\left[\begin{array}{ccc} \cos\phi & 0 & \sin\phi \\ 0 & 1 & 0\\ -\sin\phi & 0 & \cos\phi \end{array}\right]\left[\begin{array}{c} x_{3}^{\prime \prime }\\ y_{3}^{\prime \prime }\\ z_{3}^{\prime \prime } \end{array}\right]=\left[\begin{array}{c} f(m)\cos\phi +z_{3}^{\prime }\sin\phi \\ g(m)\\ -f(m)\sin\phi +z_{3}^{\prime }\cos\phi \end{array}\right]$$

This point should be on Ray 3 rotated on the x-axis.

(32) $$\left[\begin{array}{c} x_{3}^{\prime \prime \prime }\\ y_{3}^{\prime \prime \prime }\\ z_{3}^{\prime \prime \prime } \end{array}\right]=\left[\begin{array}{ccc} 1 & 0 & 0\\ 0 & \cos\theta & -\sin\theta \\ 0 & \sin\theta & \cos\theta \end{array}\right](P_{3}^{\prime }+\lambda_{3}V_{3}^{\prime })$$

Three equations are derived from the constraint of Equation (32).

(33) $$f(m)\cos\phi +z_{3}^{\prime }\sin\phi =A_{3}^{\prime }+\lambda_{3}a_{3}^{\prime }$$

(34) $$g(m)=(B_{3}^{\prime }+\lambda_{3}b_{3}^{\prime })\cos\theta -(C_{3}^{\prime }+\lambda_{3}c_{3}^{\prime })\sin\theta$$

(35) $$-f(m)\sin\phi +z_{3}^{\prime }\cos\phi =(B_{3}^{\prime }+\lambda_{3}b_{3}^{\prime })\sin\theta +(C_{3}^{\prime }+\lambda_{3}c_{3}^{\prime })\cos\theta$$

λ_3_ is expressed in terms of *m* from Equation (33). *θ* is eliminated by computing the squared sum of Equations (34) and (35). The candidates of *m* are computed by solving an eight-degree polynomial equation derived from Equations (33)-(35).

After solving the polynomial equation, we verify each solution candidate of *m* using the fact that the scale parameters λ_1_, λ_2_ and λ_3_ in Equations (4)-(6) must be positive (*i.e.*, positive depth constraint). For each candidate of *m*, first we compute λ_3_ using Equation (33) and discard the candidate if λ_3_ is negative. Next, we compute *Q*_1_ and *Q*_2_ in the canonical position of Frame 2 using Equations (18) and (19). Their z-coordinates are equal to *d*_2_. Camera centers $P_{1}^{\prime }$, $P_{2}^{\prime }$ and direction vectors $V_{1}^{\prime }$, $V_{2}^{\prime }$ in that position can be computed easily by applying the transformation *H*_2_(= [*R*_2_ *T*_2_]) to the original ones *P*_1_, *P*_2_, *V*_1_ and *V*_2_.

(36) $$P_{1}^{\prime }=R_{2}P_{1}+T_{2}$$

(37) $$P_{2}^{\prime }=R_{2}P_{2}+T_{2}$$

(38) $$V_{1}^{\prime }=R_{2}V_{1}$$

(39) $$V_{2}^{\prime }=R_{2}V_{2}$$

Scales λ_1_ and λ_2_ can be computed by Equations (4) and (5), because the relationship among *P_n_*, *V_n_* and *Q_n_* is not changed by any metric transformation. We also discard the candidate of *m* if the scales are negative. If all scales are positive, we know the coordinates of *Q*_1_, *Q*_2_ and *Q*_3_ in both frames. Their relative pose *H* is computed by registering point sets or accumulating transformations. In Equation (40), all transformations are modified into 4×4 matrices by adding fourth row [0 0 0 1] to them.

(40) $$H=\left[\begin{array}{cc} R & T\\ 0 & 1 \end{array}\right]=H_{2}^{-1}H_{4}H_{3}H_{1}$$

### 3.3. Experimental Validation

**Figure 10.**
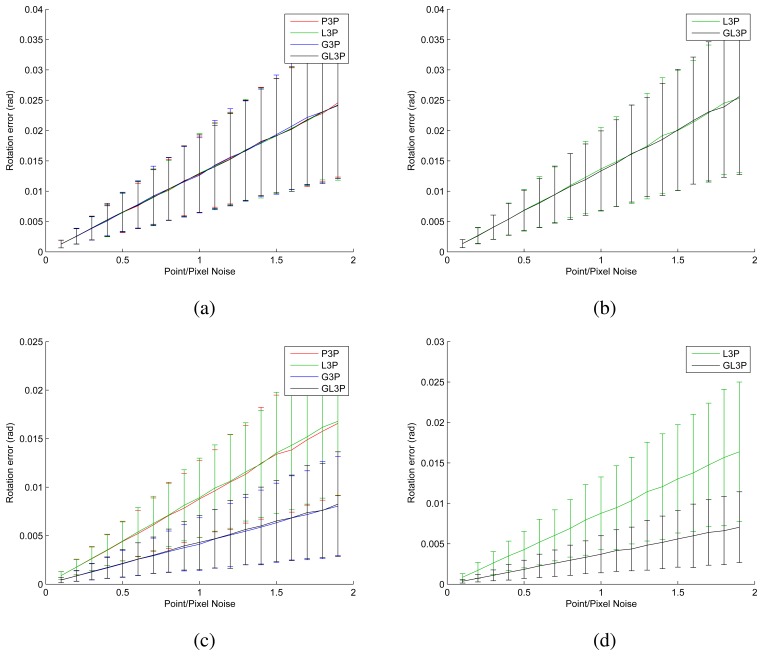
The graphs show the rotation errors of four different three-point algorithms (perspective three-point algorithm (P3P, [45]), laser three-point algorithm (L3P, [47]), generalized three-point algorithm (G3P, [46]) and generalized laser three-point algorithm (GL3P, proposed)) using synthetic data: (**a**) a single camera and non-collinear points; (**b**) a single camera and collinear points; (**c**) non-overlapping cameras and non-collinear points; and (**d**) non-overlapping cameras and collinear points. The results of P3P and G3P are not displayed in (**b**) and (**d**), because the errors resulting from them are very large (about 0.5 rad) compared to those of the other algorithms. Graphs showing translation errors are not displayed, because they resemble those showing rotation errors.

We generated a synthetic data set to verify the performance of the proposed algorithm. Three points and the relative pose are generated randomly. After generating the ground truth, we added Gaussian noise to the 3D points and projected their locations on the images. We generated data simulating single-camera system and non-overlapping two-camera system (heading in the opposite direction) using non-collinear points and collinear points. We compared four different algorithms mentioned above: perspective three-point algorithm (P3P, [45]), laser three-point algorithm (L3P, [47]), generalized three-point algorithm (G3P, [46]) and generalized laser three-point algorithm (GL3P, proposed). The simulation results displayed in Figure 10 are identical to what we expected. In the case of the two-camera setup, the generalized algorithms show better performance than the single-camera-based setup (see Figure 10c,d). We propose L3P and GL3P to avoid degeneration, and indeed, they provide more accurate results than the typical three-point algorithms in such a case (see Figure 10b,d). The proposed algorithm shows the best performance among the three-point algorithms in terms of accuracy.

We also examined the computation time of the proposed algorithm. Every three-point algorithm tested in this paper computes the solutions in three steps: (1) computes the coefficients of a four- or eight-degree polynomial equation; (2) solves the equation; and (3) computes the transformation matrix for each solution. In Table 1, ‘Equation’ and ‘Matrix’ indicate the average time for (1) + (2) and (1) + (2) + (3), respectively. Table 1 shows that the proposed algorithm computes the solution matrices faster than L3P and G3P and that its computation time is short enough to be applied to real-time implementations.

**Figure 11.**
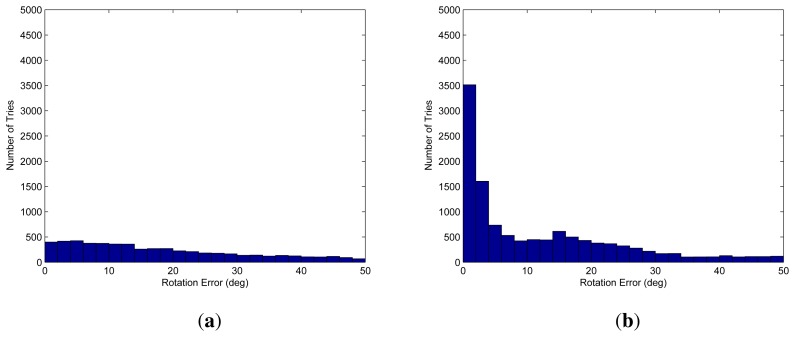
Histograms of the angle differences between the final result (i.e., refined result) and the candidates from RANSAC using the G3P algorithm [46] (**a**) and GL3P algorithm (proposed, (**b**)): The proposed GL3P algorithm provides a larger number of the accurate candidates than the G3P algorithm. Moreover, the number of the instances of degeneration cases is reduced using the proposed method; the numbers of candidates generated from 20,000 iterations are 6,650 (G3P) and 16,491 (GL3P).

The proposed algorithm is also verified using real data. Because we do not know the ground truth of the motion between the adjacent frames, the final result, which is locally refined using inliers and globally refined by loop closing, is referred to as the ground truth (*i.e.*, the desired result). We used a frame with no structures nearby to test the worst case of scanning only the ground. We selected three points randomly and computed the candidate 20,000 times using both the G3P and GL3P algorithms. The difference between the candidate and the ground truth is shown in Figure 11. The proposed method provides solutions with less serious errors than the G3P algorithm. Furthermore, the number of the samples that give no solution by the proposed method is much smaller than that by the G3P algorithm.

Although both algorithms compute an appropriate candidate with a large number of RANSAC iterations, the result shows that the proposed algorithm has a higher probability of computing an accurate initial solution within a small number of iterations.

**Figure 12.**
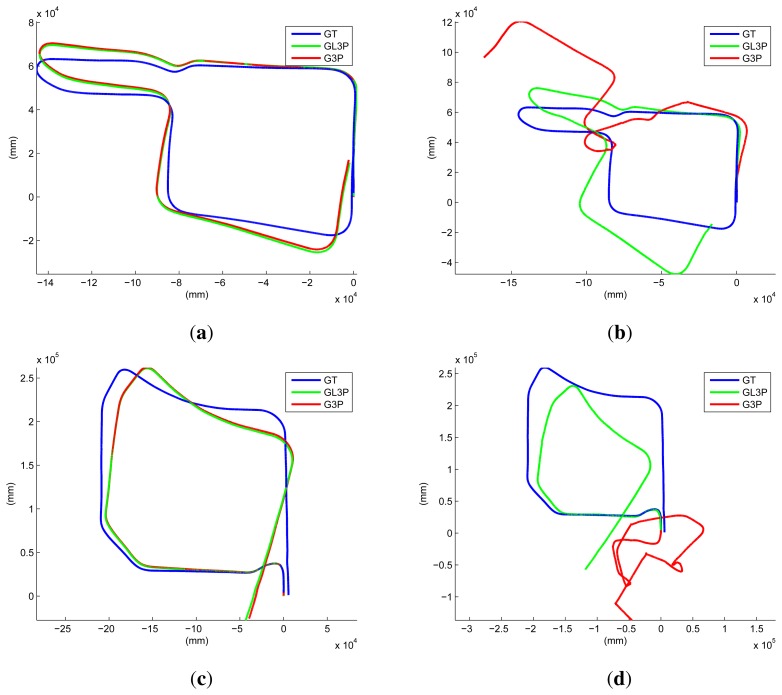
The motion of short sequences with a closed loop is estimated using the G3P [46] and the proposed GL3P algorithms. The initial motion (the result of the RANSAC process) is compared to the final result (denoted by GT (ground truth)). (**a**,**c**) Using all laser points, the results contain small accumulation errors; (**b**,**d**) When using only some of the laser points (maximum range limited to five meters), the result using the GL3P algorithm contains a small amount of drift on rotation, while the result using the G3P algorithm contains severe distortion. The lengths of the sequences are about 440 m (a,b) and 850 m (c,d).

We performed another experiment using short sequences with a closed loop. Again, we consider the final result refined using all of the inliers among the image features and the laser points as the ground truth. The results of the RANSAC process (1000 iterations) using the G3P and the proposed GL3P are compared to that result. The results are similar to the ground truth if we utilize all of the laser data (see Figure 12a,c). However, if we reduce the maximum range of the laser sensor (five meters in our experiment), both results become erroneous. In Figure 12b,d, the result using the proposed algorithm contains a small amount of drift in the rotation, while the result using the G3P algorithm contains severe distortion. When using real data, the G3P may estimate something with the laser points on the ground, because they are not perfectly collinear. However, they do not guarantee the accuracy of the estimation result using the G3P algorithm. This can be guaranteed only by the laser points on non-ground structures or with a large amount of laser data on the ground. This result shows that the proposed algorithm shows better performance than the G3P algorithm with the limitation of an actual experiment.

In order to avoid degeneration in real experiments, we recommend using long-range laser sensors to scan non-ground objects as much as possible. If there are no objects nearby, we recommend moving the system continuously (without stopping) while the system captures data. Only the proposed GL3P algorithm can provide a good initial solution in that case. Any existing algorithms and even GL3P cannot compute a proper motion if the laser sensor does not move and scans a plane. Detecting and ignoring duplicated frames at the same location may be an alternative solution for that case.

## 4. Reducing Accumulated Error

The proposed method of 3D reconstruction accumulates frame-by-frame estimation results. This type of method always experiences an error accumulation problem. This is usually solved using global sensors, such as GPS, or by closing a few loops. In this section, we present a novel method of reducing the accumulated error by utilizing a few closed loops only and not GPS. The loops to be closed are selected by a human operator without considering the additional issue of automated loop closing.

We consider the case of visiting the same place twice, while capturing data continuously. Although the local motion estimation in this case is very accurate, usually, two visits are not registered well due to accumulated error. In order to reduce the error by loop closing, the relative pose between the visits must be computed first. In this paper, this is done by the registration of 3D point clouds. The local structures of the first visit and the second visit to be registered are reconstructed using hundreds of neighboring frames. Various methods of 3D-3D registration are available, such as the ICP (iterative closest point, [48]) and EM-ICP (expectation-maximization iterative closest point, [49]) algorithms. Moreover, additional information, such as the color or template of laser points, may be utilized [23,50,51] because their projected location on images are already known.

**Figure 13.**
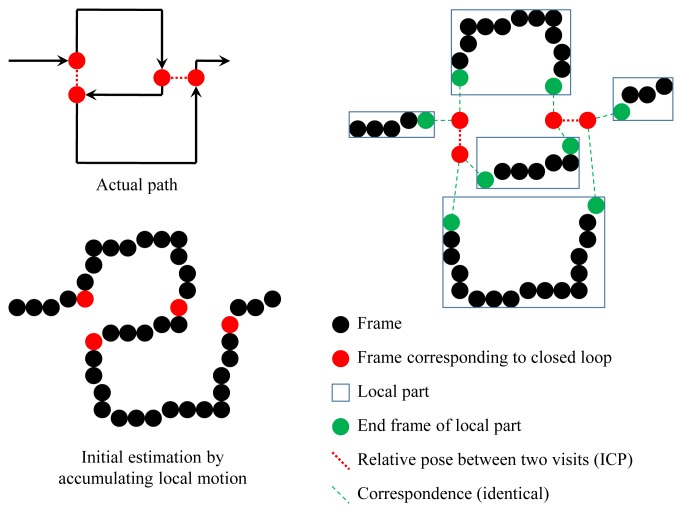
After computing the relative pose between two visits, the initial estimation is divided into local parts. In the registration process, the relative pose among the first visits of the loops (red dots) are adjusted in order to minimize the error, which should be distributed to the frames.

After the loops are closed, the error is distributed to the frames. In order to distribute the error equally and reasonably, we divide the frame-by-frame accumulation result into ‘local parts’, so that the end frames of the parts are equal to the first visit or the second visit of the closed loops, as shown in Figure 13. Let us assume that we have *N* closed loops (*i.e.*, the accumulated result is divided into 2*N* + 1 local parts). *P_i_* and *Q_i_* (*i* = 1 ∼ *N*) are the projection matrix of the first visit and relative pose between two visits of the *i*-th loop, respectively. *Q_i_* is computed by the 3D-3D registration mentioned above. We adjust the relative poses among the first visits to minimize the error to be distributed to each frame, while satisfying the closed-loop constraint. We call this process the ‘registration’ of local parts. *P*_2_ ∼ *P_N_* are the unknown variables of the registration (*P*_1_ is set to *I* as the reference coordinate). The cost function of the registration is the sum of the errors to be distributed in all parts:

(41) $$f(P_{2},\ldots ,P_{N})=\sum_{k=2}^{2N}\frac{1}{n_{k}}g(E_{k})$$

(42) $$E_{k}=\left[\begin{array}{cc} R_{k}^{E} & T_{k}^{E}\\ 0 & 1 \end{array}\right]=B_{k}A_{k}^{-1}\cdot {\left(b_{k}a_{k}^{-1}\right)}^{-1}$$

*E_k_* is the error of the *k*-th local part caused by the difference between accumulation ( $B_{k}A_{k}^{-1}$) and loop closing ( $b_{k}a_{k}^{-1}$). *A_k_* and *B_k_* are the projection matrices of the start frame and the end frame of the *k*-th local part, respectively, computed by frame-by-frame estimation. They are not changed during the registration process. *a_k_* and *b_k_* are the projection matrices of the closed-loop visits corresponding to the start frame and the end frame of the *k*-th local part, respectively. They are determined by *P_i_* and *Q_i_* (*i* = 1 ∼ *N*). If the start frame of the *k*-th local part corresponds to the first visit of the *i*-th loop, *a_k_* is set to *P_i_*. If the start frame corresponds to the second visit of the *j*-th loop, *a_k_* is set to *Q_j_P_j_*. *b_k_* is computed in the same way as *a_k_*. The weight *n_k_* is the number of frames included in the *k*-th local part. The initial value of *P_i_* is set to the projection matrix of the corresponding frame in the accumulated result.

The error function *g*(*E_k_*) consists of rotation error and translation error. Since rotation error is independent of translation error, we divide the refinement process into two steps—rotation and translation—and refine rotation matrices first. In the first step, the error function *g*_1_(*E_k_*) is defined as the rotation error computed from *R_k_*. After optimizing rotation matrices of *P*_2_ ∼ *P_N_*, rotation error *R_k_* is distributed equally to the frames included in the *k*-th local part using the method proposed by Sharp *et al.* [52], while translation vectors are fixed. After refinement of rotation matrices, translation vectors are refined by using error function *g*_2_(*E_k_*).

(43) $$g_{1}(E_{k})=\left|∠R_{k}^{E}\right|$$

(44) $$g_{2}(E_{k})=\left\|T_{k}^{E}\right\|$$

We investigated the effect of the error distribution using the ‘institute’ dataset shown in Section 5. The sequence has 23 local parts, because we utilized 12 closed loops for refinement. The rotation error and the translation error distributed to each frame are smaller than 0.001 degrees and one millimeter, respectively. For your information, the initial result (before refinement) is computed by the GL3P algorithm explained in Section 3 followed by a local refinement using inliers among image features and laser points. The closed-loop error distributed to each frame is small enough to keep the projection error of the features small. The mean value of the projection error of the laser points is increased from 0.4649 to 0.4691 (+0.59%), while that of the image features is increased from 0.2019 to 0.2060 (+2.06%). The refined result after error distribution is similar to the frame-by-frame result in terms of the projection error. The projection error is computed using the tracking result as a reference. Although the tracking result may contain some error, the process of the motion estimation minimizes the projection error using the tracking result. Hence, the error distribution process does not affect the motion estimation process significantly and allows the local estimation result to satisfy the closed-loop constraint.

## 5. Experimental Results

**Figure 14.**
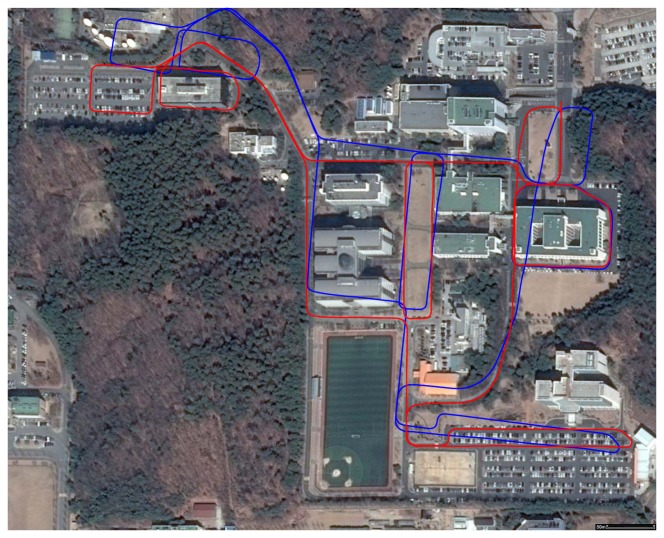
Motion estimation result of the ‘institute’ dataset. The initial result (blue) is refined (red) using 12 closed loops. The final result overlaid on the satellite image shows the accuracy of the proposed method.

**Figure 15.**
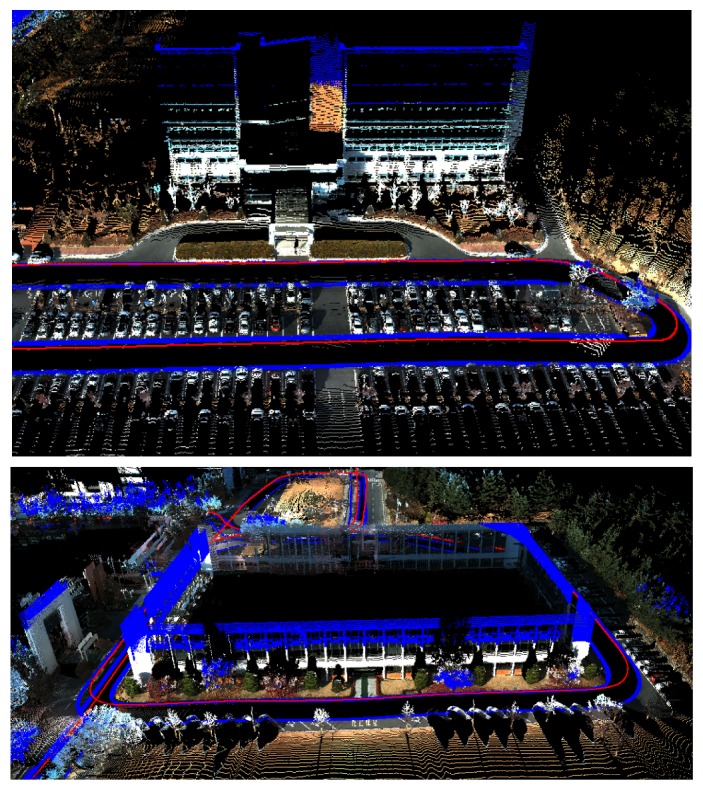
Parts of the reconstruction result of the ‘institute’ dataset. The red curve shows the path of the system. The color of the point is extracted from the images. The points out of the images are displayed as blue points.

**Figure 16.**
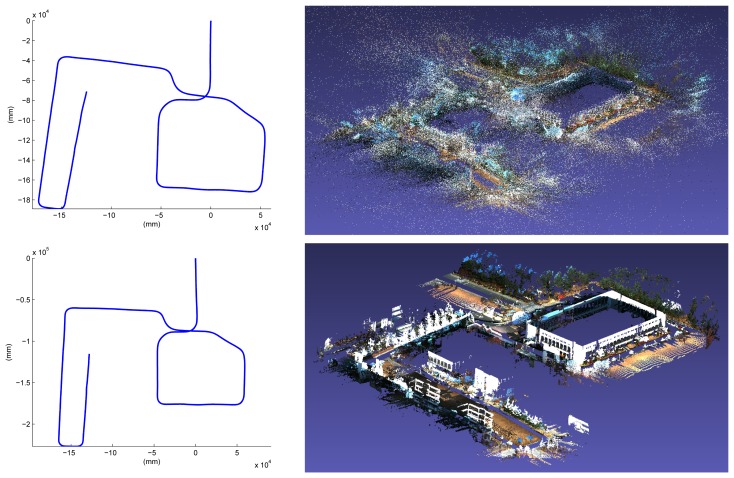
Estimated paths and reconstructed structures by a camera-based SFM (structure from motion) method ([53], **top**) and the proposed method (**bottom**).

We captured three places to verify the performance of the proposed system. The first place is a research institute in Daejeon, Korea. The result of motion estimation is shown in Figure 14. The length of the entire path is 3.5 km, and 67,300 frames were captured (‘institute’ dataset). The vehicle was driven at a low speed to obtain a dense scan result. This was possible, because there were few cars in the area. The accumulated error in the initial result is reduced by 12 closed loops. The 3D structure reconstructed by accumulating laser scans is shown in Figure 15. In order to compare the proposed method to the camera-based SFM (structure from motion) methods, we applied an SFM method [53] to the first 20,000 frames of the same dataset and reconstructed 3D structures. The results using the SFM method and the proposed method are compared in Figure 16. The camera-based SFM method computed system path and 3D structure using the triangulation results of tracked image features. The path contains some drift, and the reconstruction result is barely recognizable as an outdoor scene with a number of buildings. The proposed method computed the path and structure using both the image features and laser points. The result using the proposed method has a smaller drift on path and a small number of erroneous points. The structures are recognized easily due to the accuracy of the result.

**Figure 17.**
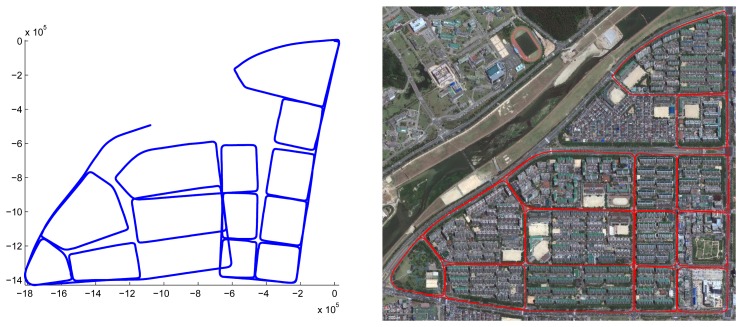
The initial estimation result (**Top**) is refined using closed loops (**Bottom**).

**Figure 18.**
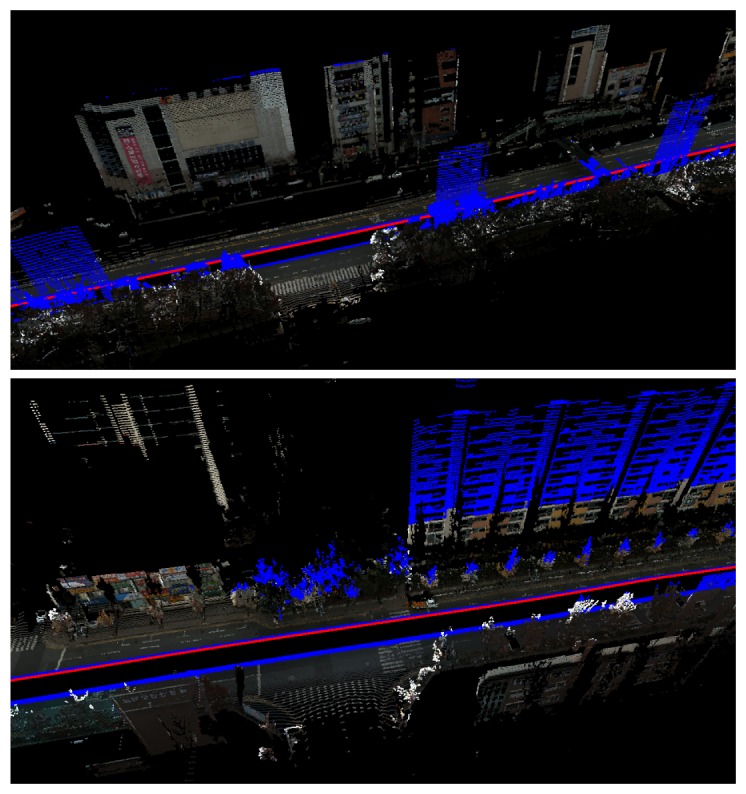
Parts of the reconstruction result of the ‘city’ dataset. The upper parts of tall buildings are displayed by blue dots, because they are not projected onto the images.

The second place is an urban part of Daejeon, Korea. We captured 14 adjacent blocks, and most roads were captured twice to generate closed loops. The length of the entire path is 20 km, and 174,000 frames were captured (‘city’ dataset). The vehicle was driven at 40 km/h, and we sometimes stopped at traffic lights, while the data were captured continuously. The upper image of Figure 17 shows the initial estimation result, which includes the accumulated error. Applying 34 closed loops, the result is refined enough to be overlaid on the satellite image (the lower image of Figure 17). The uppermost block is reconstructed and magnified in Figure 18.

**Figure 19.**
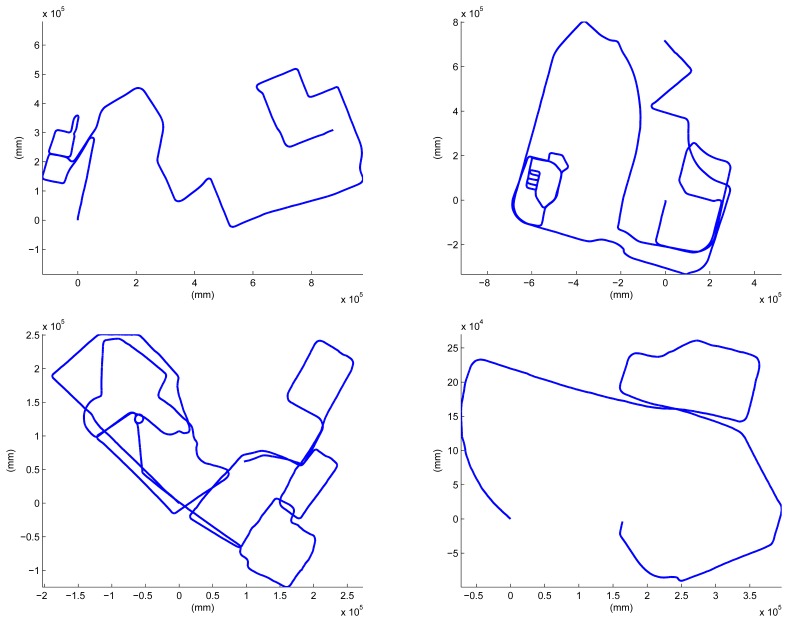
Four sequences are captured on the KAIST campus. Upper sequences are captured by the vehicle-mounted system (**Left**) 3.83 km, 42,858 frames; (**Right**) 8.27 km, 90,490 frames), and the lower sequences are captured by the hand-held system (**Left**) 3.46 km, 123,569 frames; (**Right**) 1.71 km, 60,203 frames).

**Figure 20.**
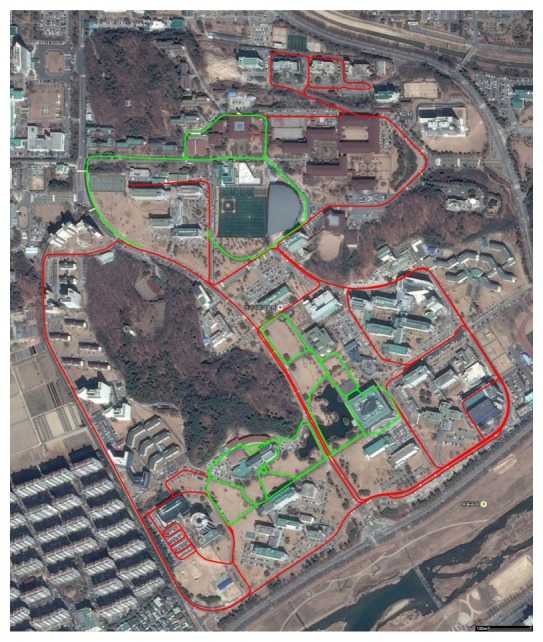
The initial results in Figure 19 are registered using 70 closed loops. The final result is successfully overlaid on the satellite image. The sequences captured by the vehicle-mounted system and the hand-held system are denoted by red and green curves, respectively.

**Figure 21.**
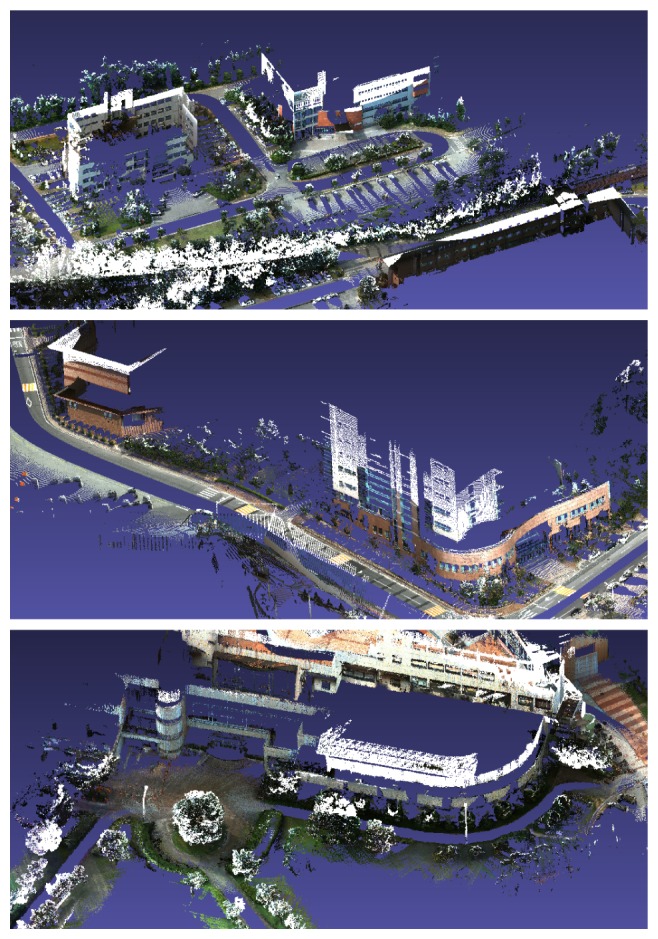
Parts of the reconstruction result of the ‘campus’ dataset.

The last place we reconstructed is the KAIST (Korea Advanced Institute of Science and Technology) campus. Most scenes contain two types of roads: for ground vehicles and those for pedestrians only. We utilized both the proposed system and the previous hand-held system in [30]. The basic rationale behind the use of both systems is ‘coarse-to-fine’. Structures near wide roads for vehicles are captured by the proposed vehicle-mounted system. Local environments in which vehicles are not allowed are reconstructed by the hand-held system. The relative pose between the results reconstructed by the different systems is computed by the registration of the local structures, as mentioned in Section 4. The method in Section 4 can be extended easily to handle multiple sequences. Two sequences are captured using the proposed system mounted on a ground vehicle, and the other two are captured using the hand-held system. The initial estimation results are shown in Figure 19. The total length of the sequence is 17.27 km, and the number of frames is 317,120 (‘campus’ dataset). The final result is overlaid on the satellite image in Figure 20. Several parts of the reconstruction result are magnified in Figure 21.

## 6. Conclusions

In this paper, we proposed a new version of a sensor fusion system of cameras and a 2D laser sensor for large-scale 3D reconstruction. The proposed system is designed to be mounted on a ground vehicle. In order to capture data when the vehicle travels at speed, we increased the frame rate of the system, so that it was higher than that of our previous system [30], and synchronized the entire system using an infrared receiver. The reconstruction is done by accumulating vertical laser scans without the assumption of 2D motion. In order to avoid degeneration when using one 2D laser sensor, we proposed a generalized laser three-point algorithm for motion estimation of the proposed system. After several loops are closed by conventional 3D-3D registration algorithms, the frame-by-frame accumulation result is adjusted slightly to satisfy the closed-loop constraint while maintaining the projection error of the features. The accuracy of the estimated path was verified by overlaying the paths on satellite images and computing the error distributed to the frames.

There are several works that could improve the proposed system and method. The proposed system uses six non-overlapping cameras to obtain a near-omnidirectional field of view. We will attempt several different arrangements of cameras to find the optimal solution. The loops are detected and closed manually in this paper, but the process can be automated using recent techniques on scene matching. Additional sensors, such as GPS and IMU, can be attached to improve the accuracy of the motion estimation.

## Figures and Tables

**Table 1.** Computation time of three-point algorithms (time unit: microseconds).

**Setup**	**Algorithm**	**Equation**	**Matrix**
Single camera	P3P	2.449	6.957
	L3P	9.080	15.382
	G3P	14.586	15.850
	GL3P	8.649	11.419

Two cameras	P3P	2.434	6.973
	L3P	9.001	14.571
	G3P	8.955	9.922
	GL3P	8.049	9.484

## References

[1] Triggs B., McLauchlan P.F., Hartley R.I., Fitzgibbon A.W. (1999). Bundle Adjustment—A Modern Synthesis. Vision Algorithms: Theory and Practice.

[2] Snavely N., Seitz S.M., Szeliski R. (2006). Photo tourism: Exploring photo collections in 3D. ACM Trans. Gr..

[3] Agarwal S., Snavely N., Simon I., Seitz S.M., Szeliski R. Building Rome in a Day.

[4] Furukawa Y., Curless B., Seitz S.M., Szeliski R. Towards Internet-Scale Multi-View Stereo.

[5] Pollefeys M., Nistér D., Frahm J.M., Akbarzadeh A., Mordohai P., Clipp B., Engels C., Gallup D., Kim S.J., Merrell P. (2008). Detailed Real-Time Urban 3D Reconstruction from Video. Int. J. Comput. Vis..

[6] Furukawa Y., Curless B., Seitz S.M., Szeliski R. Manhattan-World Stereo.

[7] Cornelis N., Leibe B., Cornelis K., Van Gool L. (2008). 3D Urban Scene Modeling Integrating Recognition and Reconstruction. Int. J. Comput. Vis..

[8] Howard A., Wolf D.F., Sukhatme G.S. Towards 3D Mapping in Large Urban Environments.

[9] Frueh C., Jain S., Zakhor A. (2005). Data Processing Algorithms for Generating Textured 3D Building Facade Meshes from Laser Scans and Camera Images. Int. J. Comput. Vis..

[10] Smith M., Posner I., Newman P. (2011). Adaptive compression for 3D laser data. Int. J. Robot. Res..

[11] Fentanes J.P., Zalama E., Gómez-García-Bermejo J. Algorithm for Efficient 3D Reconstruction of Outdoor Environments Using Mobile Robots.

[12] Banno A., Masuda T., Oishi T., Ikeuchi K. (2008). Flying Laser Range Sensor for Large-Scale Site-Modeling and Its Applications in Bayon Digital Archival Project. Int. J. Comput. Vis..

[13] Xiao J., Furukawa Y. Reconstructing the World's Museums.

[14] Allen P.K., Stamos I., Troccoli A., Smith B., Leordeanu M., Hsu Y.C. 3D Modeling of Historic Sites Using Range and Image Data.

[15] Ortín D., Neira J., Montiel J.M.M. Relocation Using Laser and Vision.

[16] Luo R.C., Lai C.C., Hsiao C.C. Enriched Indoor Environment Map Building Using Multi-Sensor Based Fusion Approach.

[17] Gallegos G., Rives P. Indoor SLAM Based on Composite Sensor Mixing Laser Scans and Omnidirectional Images.

[18] Zhang X., Rad A.B., Wong Y.K. (2012). Sensor Fusion of Monocular Cameras and Laser Rangefinders for Line-Based Simultaneous Localization and Mapping (SLAM) Tasks in Autonomous Mobile Robots. Sensors.

[19] Newman P., Cole D., Ho K. Outdoor SLAM Using Visual Appearance and Laser Ranging.

[20] McManus C., Furgale P., Barfoot T.D. Towards Appearance-Based Methods for Lidar Sensors.

[21] Newcombe R.A., Izadi H., Hilliges O., Molyneaux D., Kim D., Davison A.J., Kohli P., Shotton J., Hodges S., Fitzgibbon A. KinectFusion: Real-time Dense Surface Mapping and Tracking.

[22] Scherer S.A., Dube D., Zell A. Using Depth in Visual Simultaneous Localisation and Mapping.

[23] Whelan T., Johannsson H., Kaess M., Leonard J.J., McDonald J. Robust Real-Time Visual Odometry for Dense RGB-D Mapping.

[24] Zhang Q., Ye M., Yang R., Matsushita Y., Wilburn B., Yu H. Edge-Preserving Photometric Stereo via Depth Fusion.

[25] Barron J.T., Malik J. Intrinsic Scene Properties from a Single RGB-D Image.

[26] Yu L.F., Yeung S.K., Tai Y.W., Lin S. Shading-Based Shape Refinement of RGB-D Images.

[27] Bar-Hillel A., Hanukaev D. Fusing visual and range imaging for object class recognition.

[28] Tang J., Miller S., Singh A., Abbeel P. A Textured Object Recognition Pipeline for Color and Depth Image Data.

[29] Herbst E., Ren X., Fox D. RGB-D Flow: Dense 3-D Motion Estimation Using Color and Depth.

[30] Bok Y., Jeong Y., Choi D.G., Kweon I.S. (2011). Capturing Village-Level Heritages with a Hand-Held Camera-Laser Fusion Sensor. Int. J. Comput. Vis..

[31] Jeong Y., Bok Y., Kim J.S., Kweon I.S. Complementation of Cameras and Lasers for Accurate 6D SLAM: From Correspondences to Bundle Adjustment.

[32] Moghadam P., Bosse M., Zlot R. Line-Based Extrinsic Calibration of Range and Image Sensors.

[33] Pfaff P., Triebel R., Stachniss C., Lamon P., Burgard W., Siegwart R. Towards Mapping of Cities.

[34] Bok Y., Choi D.G., Jeong Y., Kweon I.S. Capturing City-Level Scenes with a Synchronized Camera-Laser Fusion Sensor.

[35] Bok Y., Choi D.G., Kweon I.S. Generalized Laser Three-Point Algorithm for Motion Estimation of Camera-Laser Fusion System.

[36] Kinect for Windows. http://www.microsoft.com/en-us/kinectforwindows/.

[37] MESA Imaging http://www.mesa-imaging.ch/home/.

[38] The World of pmd http://www.pmdtec.com/.

[39] Velodyne Lidar http://www.velodynelidar.com/lidar/lidar.aspx.

[40] Zhang Z. Flexible Camera Calibration By Viewing a Plane From Unknown Orientations.

[41] Shi J., Tomasi C. Good Features To Track.

[42] Lowe D.G. (2004). Distinctive Image Features from Scale-Invariant Keypoints. Int. J. Comput. Vis..

[43] Bay H., Tuytelaars T., Van Gool L. SURF: Speeded up Robust Features.

[44] Fischler M.A., Bolles R.C. (1981). Random sample consensus: A paradigm for model fitting with applications to image analysis and automated cartography. Commun. ACM.

[45] Haralick R.M., Lee C.N., Ottenberg K., Nölle M. (1994). Review and Analysis of Solutions of the Three Point Perspective Pose Estimation Problem. Int. J. Comput. Vis..

[46] Nistér D. A Minimal Solution to the Generalised 3-Point Pose Problem.

[47] Bok Y., Hwang Y., Kweon I.S. Accurate Motion Estimation and High-Precision 3D Reconstruction by Sensor Fusion.

[48] Besl P.J., McKay N.D. (1992). A Method for Registration of 3-D Shapes. IEEE Trans. Pattern Anal. Mach. Intell..

[49] Granger S., Pennec X. Multi-Scale EM-ICP: A Fast and Robust Approach for Surface Registration Robust Approach for Surface Registration.

[50] Men H., Gebre B., Pochiraju K. Color Point Cloud Registration with 4D ICP Algorithm.

[51] Tykkälä T., Audras C., Comport A.I. Direct Iterative Closest Point for Real-Time Visual Odometry.

[52] Sharp G.C., Lee S.W., Wehe D.K. (2004). Multiview Registration of 3D Scenes by Minimizing Error between Coordinate Frames. IEEE Trans. Pattern Anal. Mach. Intell..

[53] Mouragnon E., Lhuillier M., Dhome M., Dekeyser F., Sayd P. Real Time Localization and 3D Reconstruction.

